# Single-Case Experimental Designs to Evaluate Novel Technology-Based Health Interventions

**DOI:** 10.2196/jmir.2227

**Published:** 2013-02-08

**Authors:** Jesse Dallery, Rachel N Cassidy, Bethany R Raiff

**Affiliations:** ^1^University of FloridaDepartment of PsychologyGainesville, FLUnited States; ^2^Rowan UniversityDepartment of PsychologyGlassboro, NJUnited States

**Keywords:** Research design, technology, mHealth, single-case design, preliminary efficacy

## Abstract

Technology-based interventions to promote health are expanding rapidly. Assessing the preliminary efficacy of these interventions can be achieved by employing single-case experiments (sometimes referred to as n-of-1 studies). Although single-case experiments are often misunderstood, they offer excellent solutions to address the challenges associated with testing new technology-based interventions. This paper provides an introduction to single-case techniques and highlights advances in developing and evaluating single-case experiments, which help ensure that treatment outcomes are reliable, replicable, and generalizable. These advances include quality control standards, heuristics to guide visual analysis of time-series data, effect size calculations, and statistical analyses. They also include experimental designs to isolate the active elements in a treatment package and to assess the mechanisms of behavior change. The paper concludes with a discussion of issues related to the generality of findings derived from single-case research and how generality can be established through replication and through analysis of behavioral mechanisms.

## Introduction

The field of technology-based behavioral health interventions is expanding rapidly. New technologies are enabling access to, and assessment of, individuals and their health-related behavior [1-3]. Even “old” technology, such as the Internet and mobile phones, is being harnessed in new ways, delivering state-of-the-art behavior therapy across diverse settings [4-7]. The fields of eHealth, mHealth, and the promise of emerging technologies have the potential to transform many systems of health care and improve public health by increasing access to cost-effective interventions. With these opportunities comes the need to evaluate rigorously the potential efficacy of new treatments. In this paper, we describe some challenges and methodological solutions associated with testing preliminary efficacy. In particular, we focus on the solutions offered by single-case experiments, which fill a unique and vital niche in the ecology of research designs. We also highlight advances in developing and evaluating single-case experiments, which help ensure that treatment outcomes are reliable, replicable, and generalizable. Finally, we describe experimental designs that allow researchers to isolate the active elements in a treatment package and to assess the mechanisms of behavior change. Our goal is to introduce a range of techniques that will be relevant to behavioral scientists that are unfamiliar with single-case research and that are particularly well suited for the research and development of new technology-based interventions. We hope to supply enough detail to achieve a basic understanding of the mechanics, utility, and versatility of single-case research and enough resources to propel further inquiry.

Broadly, single-case designs include a family of methods in which each participant serves as his or her own control. In a typical study, some behavior or self-reported symptom is measured repeatedly during all conditions for all participants. The experimenter systematically introduces and withdraws control and intervention conditions and then assesses effects of the intervention on behavior across replications of these conditions within and across participants. Thus, the telltale traits of these studies include repeated and frequent assessment of behavior, experimental manipulation of the independent variable, and replication of effects within and across participants. Although some forms of replication are readily apparent, such as replications of effects within and between subjects, other forms may be more subtle. For example, replication within subjects also occurs by simply measuring behavior repeatedly within a condition. Assuming some degree of stability of the dependent variable within a condition, there will be many replications of the effects of a treatment on behavior.

A recent study illustrates the efficiency and rigor of a single-case design to assess a novel technology-based treatment [8]. Raiff and Dallery assessed whether an Internet-based incentive program could increase adherence to blood glucose testing for 4 teenagers diagnosed with Type 1 diabetes. Teens monitored glucose levels with a glucose meter during a 5-day baseline (control) condition. During a 5-day treatment condition, participants earned vouchers (statements of earnings exchangeable for goods and services) for adhering to blood glucose testing recommendations (ie, 4 tests per day). After the treatment condition, participants monitored blood glucose just as they did during the first baseline condition for 5 days, without the possibility of earning incentives. Participants submitted a mean of 1.7 and 3.1 blood glucose tests per day, respectively, during the baseline and return-to-baseline conditions, compared to 5.7 tests per day during the treatment condition. Because adherence increased only when the treatment was implemented for all 4 participants and because behavior within each condition was stable (ie, five replications of treatment effects per participant and ten replications of control levels per participant), this experiment suggested that an Internet-based incentive program can reliably increase adherence to self-monitoring of blood glucose.

## A Symbiosis Between Single-Case Designs and Technology-Based Data Capture

We believe that a symbiosis exists between single-case experiments and technology-based interventions. Single-case designs can capitalize on the ability of technology to easily, unobtrusively, and repeatedly assess health-related behavior [7,9]. Single-case research requires frequent contact with the participant’s behavior, which can be challenging in some research contexts but is more straightforward with technology. For example, researchers have used technology-based measures of activity in the form of daily step counts [10], twice-daily measurements of exhaled carbon monoxide as an indicator of smoking status [11], and medication adherence on a daily basis [12]. Assessment may become even easier as unobtrusive biometric sensors “weave themselves into the fabric of everyday life until they are indistinguishable from it” [13] [2,14]. Such repeated assessment, whether through existing or new technology, provides excellent opportunities to analyze the effects of treatment variables using single-case experiments. In addition, many technology-delivered behavioral health interventions permit automated treatment delivery [15]. This means that treatment can be delivered with high fidelity, which can minimize between-subject variability in treatment dose and quality. Because detecting treatment effects in single-case designs requires replications across subjects, ensuring equivalent treatment fidelity and quality across participants enhances the internal validity of the study.

There are two additional advantages of single-case research, and these advantages exist whether patient improvement is measured with technology-based or alternative methods. First, because “health” is a property of an individual (and not a group of individuals), assessing change over time in an individual patient’s behavior is an empirical and conceptual necessity. Single-case research requires a fine-grained view of health-related behavior over time, and technology-based data capture can enable this view. Second, single-case research is also well suited to demonstrate preliminary efficacy, which can be defined as “clinically significant patient improvement over the course of treatment” [16]. Patient improvement can be revealed by changes in health-related behavior from baseline to treatment, and the cause of these changes can be verified via replications within and across participants. Experimental designs, such as group designs (cf. [17]) that take only a “snapshot” of behavior, fail to resolve this temporally dynamic feature of behavior. As noted by Morgan and Morgan [18], this failure is “equivalent to underusing the resolving power of a microscope.”

In addition to the fit between the logic of single-case designs and the data capture capabilities of technology, single-case designs may obviate some logistical issues in using between group designs to conduct initial efficacy testing. For example, prototypes of a new technology may be expensive and time consuming to produce [1]. Similarly, troubleshooting and refining the hardware and software may entail long delays. For these reasons, enrolling a large sample for a group design may be prohibitive. Also, during development of a new technology-based treatment, a researcher may be interested in which components of treatment are necessary. For example, a mobile-phone based treatment may involve self-monitoring, prompts, and feedback. Assessing these components using a group design may be cumbersome. Single-case designs can be used to perform efficient, systematic component analyses [19]. Although some logistical issues may be mitigated by using single-case designs, they do not represent easy alternatives to traditional group designs. They require a considerable amount of data per participant (as opposed to a large number of individuals in a group), enough participants to reliably demonstrate experimental effects, and systematic manipulation of variables over a long duration. Nevertheless, in many cases, single-case designs can reduce the resource and time burdens associated with between group designs.

## Addressing Common Misconceptions

There are several common misconceptions about single-case designs [20,21]. First, single-case does not mean “n of 1”. The number of participants in a typical study is always more than 1, usually around 6 but sometimes as many as 20, 40, or more participants [11,22]. Also, the unit of analysis, or “case”, could be individual participants, clinics, group homes, hospitals, or health care agencies. Given that the unit of analysis is each case, a single study could be conceptualized as a series of single-case experiments. Second, single-case designs are not limited to interventions that produce large immediate changes in behavior. They can be used to detect small but meaningful changes in behavior and to assess behaviors that may change slowly over time (eg, learning a new skill) [23]. Third, findings from single-case research do not inherently lack external validity or generality. This misconception is perhaps the most prejudicial, and addressing it requires some background in the logic and mechanics of single-case design. Thus, we shall save our discussion of this misconception to the end of this paper.

## Structures and Functions of Single-Case Designs

The most common single-case designs—and those that are most relevant to technology-based interventions—are presented in Table 1. The table also presents some procedural information, as well as advantages and disadvantages for each design. All of these designs permit inferences about causal relations between independent and dependent variables (observations of behavior, self-reports of symptoms, etc). Procedural controls must be in place to make these inferences such as clear, operational definitions of the dependent variables, and reliable and valid techniques to assess the behavior. The experimental design must be sufficient to rule out alternative hypotheses for the behavior change. Table 2 presents a summary of the main methodological and assessment elements that must be present to permit conclusions about treatment effects [24]. The majority of the criteria in Table 2 have been validated to evaluate the quality of single-case research [25]. As such, the items listed in the table represent quality control standards for single-case research.

We have added one criterion to Table 2, that is, researchers should authenticate the participant who generated the dependent variable or use validation methods to assess whether the participant (and not some other person) was the source of the data. Authentication or validation is important when data capture occurs remotely with technology. The difficulty in ensuring that remote sensors are collecting data about a specific individual is referred to as the “one body authentication problem” [26]. To solve this problem, for example, a web-based video [7] or new methods in biometric fingerprinting could authenticate the end-user [26,27]. As an alternative, or as a complement, validation measures can be collected. For example, in-person viral load assessments could be measured at various points during a study to increase antiretroviral medication adherence [12], or body mass and physiological measures could be measured during an exercise or activity-based intervention.

There are two additional assessment-related items in Table 2 that warrant discussion in the context of novel technology-based interventions. The first is assessing the fidelity of technology-based treatments [28]. Carroll and colleagues [29] defined fidelity ‘‘as the degree to which the intervention implementation process is an effective realization of the intervention as planned’’ (p. 1). This definition entails measurement of the delivery and receipt of the intervention, which are related but not necessarily synonymous. What is delivered via technology may not be what is received by the end-user. Dabbs and associates [28] provide a list of questionnaire items that could be easily adapted to assess the fidelity of technology-based interventions. These items are based on the Technology Acceptance Model [30]. The second is assessing whether the methods and results are socially valid [31,32]; see Foster and Mash [33] for methods to assess social validity. Social validity refers to the extent to which the goals, procedures, and results of an intervention are socially acceptable to the client, the clinician or health care practitioner, and society [33-37]. During initial efficacy testing, social validity from the perspective of the client should be assessed. Indeed, technology may engender risks to privacy and confidentiality, and even an effective intervention may be perceived as too intrusive.

**Table 1:** Common single-case designs, including general procedures, advantages, and disadvantages.

Design	Procedure	Advantages	Disadvantages
Reversal	Baseline conducted, treatment is implemented, and then treatment is removed	Within-subject replication; clear demonstration of an intervention effect in one subject	Not applicable if behavior is irreversible, or when removing treatment is undesirable
Multiple-Baseline	Baseline is conducted for varying durations across participants; then treatment is introduced in a staggered fashion	Treatment does not have to be withdrawn	No within-subject replication; potentially more subjects needed to demonstrate intervention effects than when using reversal design
Alternating Treatment	Baseline and multiple different treatments are quickly alternated (often within the same day)	Within-subject replication; rapid demonstration of differences between several treatments	Sequence effects (ie, treatment interaction) can occur; phases may be difficult to discriminate if changed too rapidly
Changing Criterion	Following a baseline phase, treatment goals are implemented; goals become progressively more challenging as they are met	Demonstrates within-subject control by levels of the independent variable without removing treatment; useful when gradual change in behavior is desirable	Not applicable for binary outcome measures; must have continuous outcomes
Combined	Elements of any treatment can be combined.	Allows for more flexible, individually tailored designs	If different designs are used across participants in a single study, comparisons across subjects can be difficult

**Table 2:** Quality indicators for single-case research.

**Dependent variable**	
	Dependent variables are described with operational and replicable precision
	Each dependent variable is measured with a procedure that generates a quantifiable index
	Dependent variables are measured repeatedly over time
	In the case of remote data capture, the identity of the source of the dependent variable should be authenticated or validated
**Independent variable**	
	Independent variable is described with replicable precision
	Independent variable is systematically manipulated and under the control of the experimenter
	Overt measurement of the fidelity of implementation of the independent variable is highly desirable
**Baseline**	
	The majority of single-case research will include a baseline phase that provides repeated measurement of a dependent variable and establishes a pattern of responding that can be used to predict/compared against the pattern of future performance, if introduction or manipulation of the independent variable did not occur.
	Baseline conditions are described with replicable precision.
**Experimental Control/Internal Validity**	
	The design provides at least three demonstrations of experimental effect at three different points in time.
	The design controls for common threats to internal validity (eg, permits elimination of rival hypotheses).
	There are a sufficient number of data points for each phase (eg, minimum of five) for each participant.
	The results document a pattern that demonstrates experimental control.
**Social Validity**	
	The dependent variable is socially important.
	The magnitude of change in the dependent variable resulting from the intervention is socially important.
	The methods are acceptable to the participant.

### General Characteristics of Single-Case Designs

Of the designs listed in Table 1, the reversal, multiple-baseline, and changing criterion designs may be most applicable for initial efficacy testing of technology-based interventions. All of these designs entail a baseline period of observation. During this period, the dependent variable is measured repeatedly under control conditions, for example for several days. Ideally, the control conditions should include all treatment elements (eg, access to the Internet, the use of a mobile phone, or technology-based self-monitoring) except for the active treatment ingredients [38]. For instance, Dallery and colleagues used a reversal design to assess effects of Internet-based incentive program to promote smoking cessation, and the baseline phase included self-monitoring, video-based carbon monoxide confirmation via a web camera, and monetary incentives [11]. The active ingredient in the intervention, incentives contingent on objectively verified smoking abstinence (via video), was not introduced until the treatment phase. An additional consideration in the context of technology is the time needed to simply learn how to operate the device, website, or software. Baseline control conditions may need to take this learning into account before the active ingredients of the intervention are introduced. The baseline condition in the study by Dallery et al, for example, provided ample time for the participants to learn how to upload videos and navigate the study website.

The duration of the baseline should be sufficient to predict future behavior. That is, the level of the dependent variable should be stable enough to predict its direction if the treatment were not introduced. If there is a trend in the direction of the anticipated treatment effect during baseline, the ability to detect a treatment effect will be limited. Thus, stability, or trend in the direction opposite the predicted treatment effect, is desirable. The decision to change conditions is an experimenter decision, which can be supplemented with a priori stability criteria [39-41]. For example, a decision to change conditions could be made if the first two and last two data points in a five-session block are within 15% of each other, and there are no visual trends in the direction of the treatment effect as determined by two independent experimenters or by a regression coefficient above or below a certain threshold. There are no universal rules about specific criteria; they must be developed in consideration of the behavior and intervention being studied.

### Reversal Designs

In a reversal design, the treatment is introduced after the baseline period. The number of data points in the treatment condition must again be sufficient to predict behavior if treatment were to continue (eg, stable performance and no trends toward baseline levels of the dependent variable). Following the treatment period, the baseline period is re-introduced, hence the “reversal” in this design. The minimum number of alternations to document experimental control in a reversal design is three alternations. Using only two conditions, such as a pre-post design, is not considered sufficient to demonstrate experimental control because other sources of influence over behavior cannot be ruled out [42]. For example, a smoking cessation intervention could coincide with a price increase in cigarettes. By returning to baseline conditions, we could assess and possibly rule out the influence of the price increase on smoking. Researchers also often employ a “reversal” to the treatment condition. Thus, the experiment ends during a treatment period. Not only is this desirable from the participant’s perspective, it provides a replication of the main variable of interest, ie, the treatment [39,43].

**Figure 1:**
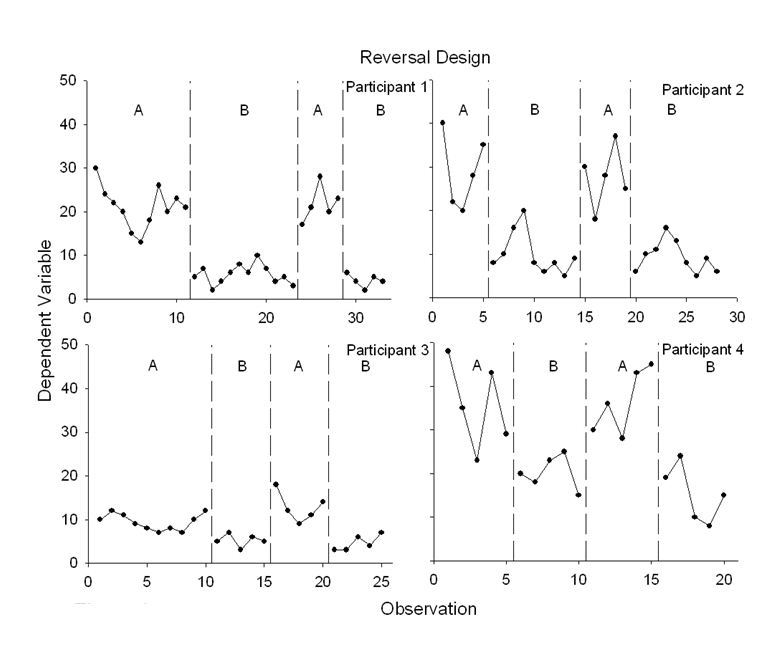
Example of a reversal design showing experimental control and replications within and between subjects (each panel represents a different participant, each of whom experienced two baseline and two treatment conditions).

Figure 1 displays an idealized, four-condition reversal design, and each panel shows data from a different participant. For the purposes of illustration, let us assume that the treatment is a text-message system delivered via mobile phone to decrease smoking (labeled “B” in the Figure). The baseline control conditions (labeled “A” in the Figure) include neutral text messages (ie, texts that are not smoking-related). Let us also assume that the dependent variable is number of cigarettes smoked per day. Although all participants were exposed to the same four conditions, the duration of the conditions differed because of trends in the conditions. For example, for Participant 1 the beginning of the first baseline condition displays a consistent downward trend (in the same direction as the expected text-message treatment effects). If we were to introduce the smoking cessation–related texts after only 5 or 6 baseline sessions, it would be unclear if the decrease in smoking was a function of the independent variable. Therefore, continuing the baseline condition until there is no visible trend helps build our confidence about the causal role of the treatment when it is introduced. The immediate decrease in the level of smoking for Participant 1 when the treatment is introduced also implicates the treatment. We can also detect, however, an increasing trend in the early portion of the treatment condition. Thus, we need to continue the treatment condition until there is no undesirable trend before returning to the baseline condition. Similar patterns, which also illustrate differences in the magnitude and variability of the effects, can be seen for Participants 2-4.

### Multiple-Baseline Design

**Figure 2:**
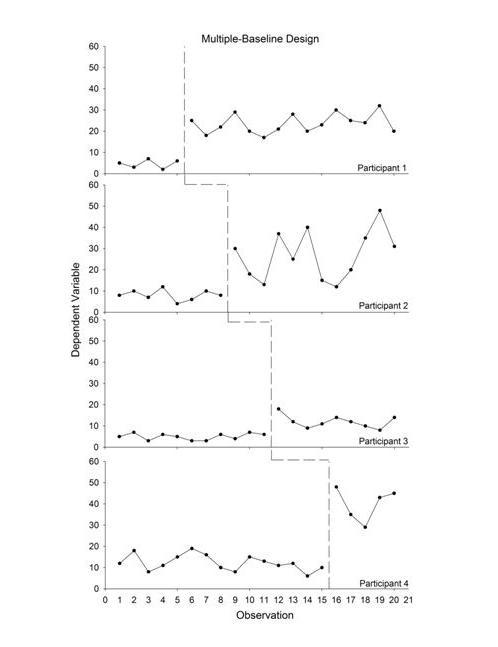
Example of a multiple baseline design showing experimental control and replications between subjects (each row represents a different participant, each of whom experienced a baseline and treatment; the baseline durations differed across participants).

In a multiple-baseline design, the durations of the baselines vary systematically for each participant in a so-called “staggered” fashion. For example, 1 participant may start treatment after 5 baseline days, another after 7 baseline days, then 9, and so on. After baseline, treatment is introduced and it remains until the end of the experiment (ie, there are no reversals). These designs are also referred to as “interrupted time-series” designs [44]. The power of these designs is derived from demonstrating that change occurs when, and only when, the intervention is directed at a particular participant (or whatever the unit of analysis happens to be [45]). The influence of other factors, such as idiosyncratic experiences of the individual or self-monitoring (eg, reactivity), can be ruled out by replicating the effect across multiple individuals. As replications are observed across individuals and behavior changes when, and only when, treatment is introduced, confidence that behavior change was caused by the treatment increases. These designs are also useful for technology-based interventions that teach new skills, where behavior would not be expected to “reverse” to baseline levels. Multiple-baseline designs also obviate the ethical concern that control participants in a between group design are not exposed to the active treatment, as all participants are exposed to the (potentially) active treatment with multiple-baseline designs. Although all participants in a reversal design also receive the treatment, the treatment must be withdrawn to assess treatment effects. Figure 2 illustrates a simple, two-condition multiple-baseline design replicated across 4 participants. Similar to the reversal design, treatment should be introduced only when the data appear stable. The durations of the baseline conditions are staggered for each participant, and the dependent variable increases when, and only when, the independent variable is introduced for all participants. Figure 2 suggests reliable increases in behavior and that the treatment was responsible for these changes.

The multiple-baseline is an advance over pre-post post designs, which also involve a baseline (or pre-intervention) period followed by a treatment period [42]. Although pre-post designs have been used to establish the feasibility of technology-based interventions [46], one advantage of using a multiple-baseline design is that in addition to establishing feasibility, it can establish preliminary efficacy [47]. For example, Cushing, Jensen, and Steele [48] investigated the ability of a mobile device, used to measure adherence to a self-monitoring intervention, to improve weight management with a multiple-baseline design. Overweight adolescents (n=3) were given weekly self-monitoring goals based on recording their meals and activity levels. During baseline, self-monitoring was completed with a traditional pencil-and-paper method, and goal attainment was measured for 4, 5, and 9 weeks for each successive participant. Following baseline, participants were instructed to use mobile devices with automated software to input their daily health information. Goal attainment increased dramatically when the mobile device was used, and the staggered presentation of the independent variable convincingly demonstrated that the mobile device increased self-monitoring of food intake and activity levels, as opposed to some other variable.

### Changing Criterion Design

The changing criterion design is also relevant to testing technology-based interventions. In a changing criterion design, a baseline is conducted until stability is attained. Then a treatment goal is introduced, and goals are made progressively more difficult. Behavior should track the introduction of each goal, thus demonstrating control by the level of the independent variable [39,45]. For example, Kurti and Dallery [10] used a changing criterion design to increase activity in 6 sedentary adults using an Internet-based contingency management program to promote walking. Weekly step count goals were gradually increased across 5-day blocks. The step counts for all 6 participants increased reliably with each increase in the goals, thereby demonstrating experimental control of the intervention.

## Analytic Techniques to Isolate Treatment Effects

The first and most important analysis of whether a technology-based intervention affected a health-related behavior is visual analysis of the time-series data. Clinically significant change in patient behavior should be visible. Even a change in a slowly developing skill should be visible in the graphical display of the data. Visual analysis prioritizes *clinically* significant change in health-related behavior as opposed to *statistically* significant change in group behavior [16]. A statistically significant effect may be clinically meaningless [49]. These observations are not new—they echo repeated calls to reform analytic strategies in psychological science [49-51]. These calls have also emphasized graphical design and visual analysis as vital elements of data analysis. Decisions about whether the magnitude of change is clinically relevant should not be automated; they should be based on visual analysis, experience with the subject matter, and scientific judgment. For example, the data in Figure 1 may suggest a reliable change in cigarettes smoked per day. Whether such reductions are meaningful, however, is another issue that can be informed by previous research on the extent to which reductions in smoking result in reductions in health risks or future smoking cessation [52].

Parsonson and Baer described several heuristics for evaluating changes in the time-series of behavioral data [53]. Several features of the data paths under each condition must be evaluated. Single-case designs use “steady-state” design logic, which at a minimum entails a stable baseline. Ensuring a sufficiently long and stable baseline permits prediction of behavior if an intervention is not introduced (see Table 2). Although the precision duration is determined by the experimenter in consideration of the dynamics of the behavior being studied, the presence of reactivity, and so on, a rule of thumb is a minimum of five data points to detect stability or trends in the data [24]. When the intervention is introduced, a large change in level (change in behavior from the last data point in baseline to the subsequent data point in treatment) and a large change in the mean (average levels in both conditions) increases confidence that experimental control was achieved. We also consider the overall pattern in the results, the amount of variability within and between phases, and the number of replications of effects both within (if the design permits it) and across participants. These heuristics highlight the power of visual analysis to simultaneously assess a number of data attributes, such as the immediacy of treatment effects, variability within and across conditions, trends, and whether the whole data series corresponds to the effects predicted by the intervention and study design [54]. To our knowledge, no other analytic technique can accomplish these tasks simultaneously.

New aids have been developed to assist in the visual analysis of time-series data [23]. One particularly powerful aid, called the conservative dual-criteria (CDC) method, helps the analyst judge whether a treatment effect is present relative to a baseline condition [55]. Essentially, the CDC method entails extending regression lines based on baseline performance into the treatment phase. The regression lines represent predictions of the data path if the intervention had not been introduced. The number of data points above (or below, depending on the predicted treatment effect) the lines are counted, and the binomial formula is used to assess whether this number exceeds what would be expected by chance. Monte Carlo simulations showed that the CDC method had acceptable rates of Type I error even with small datasets [55]. Furthermore, the method had greater power than other common aids to visual analysis, such as the split-middle method, and outperformed two common statistical methods to analyze time-series data (interrupted time series, general linear model), even with the presence of autocorrelation [55].

Before statistical tests are applied, the presence of autocorrelation in the time-series data must be considered. Autocorrelation means that many traditional parametric and nonparametric tests may not be appropriate to analyze treatment effects (eg, *t*, *F*, chi square, etc). Autocorrelation is when successive data points are correlated, for example, mood on day 1 is correlated with mood on day 2, and so on. The presence of autocorrelation can be assessed by calculating an autocorrelation coefficient. There is disagreement about how much autocorrelation occurs in single-case time-series data and the extent to which it inflates Type I error rate [53,55]. At a minimum, the issue of autocorrelation must be considered when deciding which statistical test is appropriate. Bockhardt and colleagues [56] framed the issue nicely:

Though it is a statistical nuisance, by its nature serial dependence reflects the momentum and gradualism of physiological, behavioral, and emotional repair. Because it is an index of serial dependence, autocorrelation can reveal something about the ebb and flow of behavioral change over time. For this reason, autocorrelation is the natural subject matter of a behavioral science. Whatever inferential statistic is applied to single-case time-series data, we believe it should approach autocorrelation not as noise that obscures change, but as music that accompanies it. Put differently, the preferred statistic gauges the occurrence of change, while preserving its structure.

There are a number of statistical techniques that can control for the presence of autocorrelation when assessing treatment effects. Although a complete discussion of these techniques is beyond the scope of this paper, several regression-based approaches are available, such as autoregressive models, robust regression, and hierarchical linear modeling (HLM) [57,58]. One limitation of some of these approaches is that they require long data streams (eg, 30 data points per condition). At least one study, however, suggests that HLM may be used with the shorter data streams seen in typical single-case studies [59] and that are consistent with the standards presented in Table 2 (ie, a minimum of five data points per condition with no undesirable trends). HLM has also been used to assess data streams collected with technology-based methods. For example, Ben-Zeev and colleagues [60] used handheld personal digital assistants to collect data about persecutory ideation in individuals diagnosed with schizophrenia and HLM to assess relations between negative affect and persecutory ideation.

Methods for computing effect sizes in single-case research have also proliferated. These methods are a welcome advance, particularly in consideration of efforts to reform traditional null hypothesis significance testing and replace *P* values with more informative effect size estimates and confidence intervals [49,61]. Parker and Hagan-Burke [62] note that effect sizes in single-case research provide: (1) an objective measure of intervention strength (assuming a strong, internally valid design), (2) a continuously scaled index to support incremental treatment decisions, (3) improved measurement precision when results are not large and obvious, (4) an objective summary when visual judgments do not agree, (5) a method for comparing relative intervention success across single-case studies, both at the local level and within broader meta-analyses, (6) improved credibility for single-case studies in the eyes of other research traditions, and (7) an efficient method of documenting results.

**Figure 3:**
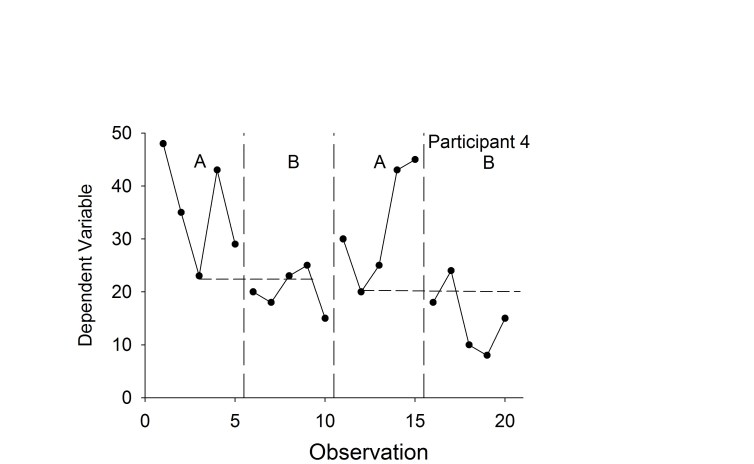
A visual example of how to calculate the percentage of nonoverlapping data (see text for calculations).

One family of effect size measures is called nonoverlap techniques. In nonoverlap calculations, the degree of nonoverlap in the data between phases (ie, between two distributions) is compared. For example, consider the bottom right panel of Figure 1, which is re-drawn as Figure 3. One basic technique is to draw a horizontal line at the lowest baseline data point because the intervention sought to decrease behavior [63]. Then, the proportion of data points below this line is calculated for the following “B” phase (eg, 3/5 or 60%). Because treatment effects were replicated, the numbers are summed from the two conditions (eg, (3 of 5) + (4 of 5), or 7 of 10 = 70%). Nonoverlap methods accord nicely with visual analysis, as one key task in visual analysis is detecting the degree of difference (nonoverlap) in the data points across successive conditions. Further, nonoverlap methods provide meaningful information about treatment effects. Nonoverlap scores above 90% are very effective, 70-90% are effective, 50-70% are questionable, and below 50% suggests the treatment was ineffective [63]. The summary measures derived from nonoverlap techniques can be used to compare different treatments for the same problem in meta-analyses.

There are other effect size calculation techniques in addition to nonoverlap methods. Manalov and colleagues [54] compared the performance of four techniques using Monte Carlo simulations. Potential confounding variables were also introduced such as autocorrelation, linear and curvilinear trends, and heteroscedasticity between conditions. Although they found that the different techniques performed better or worse depending on the nature of the data, one overlap technique called nonoverlap of all pairs performed adequately across all conditions. The authors presented a simple flowchart for decision making to select an effect size technique based on the properties of the data (eg, the presence of linear trend).

Due in part to the recent advances in statistical and effect size calculations, meta-analysis of single-case studies have started to appear in the literature. Several meta-analyses have used a variant of the nonoverlap technique described above as a measure of effect size (see [64] for details about this technique) [65-67]. Other researchers have used HLM to perform meta-analysis [68]. Jenson and colleagues [59] conducted Monte Carlo simulations of reversal designs using HLM with different amounts of autocorrelation, data points, and effect sizes and found that HLM performed well (eg, Type I error rates were acceptable). Also, under the vast majority of conditions HLM produced power greater than 0.9. In only 8 out of the 30 conditions did power drop below this number, and these conditions included small numbers of data points in baseline and treatment conditions combined with high amounts of autocorrelation (>0.8, indicating a strong trend). In light of the quality control criteria presented in Table 2, these conditions should be rare in published single-case studies.

To our knowledge, with the exception of HLM, many of the techniques described above have not been applied to assess effects of technology-based interventions. This is not surprising, as both the statistical methods and technology-based interventions are relatively new. With respect to statistical analyses of time-series data, the number of techniques have proliferated in recent years; some authors estimate that the number has tripled since the 1980s (Parker et al, 2005). One potential negative side effect of such proliferation is the lack of standards or rules to guide decision making about appropriate statistical tests. Thus, we recommend Kazdin’s [23] or Barlow and colleague’s [45] textbooks as useful resources regarding statistical analysis of time-series data. But, we hasten to note that statistical analysis should be viewed as a complement to visual analysis, not a replacement. As noted by Kazdin [23]:

We would like simple rules to guide us and to teach our students. We have a couple, perhaps: (1) consider more than one means of evaluating the data, and (2) in relation to visual inspection and statistical analysis, do not take an “either/or” position. Either/or may work well in philosophy (Kierkegaard, 1843), but may not be wise in science.

## Some Threats to Inferences Based on Single-Case Designs

There are several potential threats to internal validity when using single-case designs. First, behavioral reactivity to the mere act of measuring behavior may be present during initial observations. Continued or unobtrusive observation can remedy this problem [45]. Given the remote data capture inherent in many technology-based interventions (eg, telemetric monitoring; [9]), reactivity may be minimized. Second, carryover effects from condition to condition may occur when using a reversal design or an alternating treatment design (in which conditions alternate more rapidly than in a reversal design; see Table 1). One solution is to specifically assess order effects by manipulating the order of sequences across participants. Another is to increase the duration of conditions: carryover effects are typically transient and will generally decrease with extended contact with the new condition. Several researchers have even recommended randomization to treatment order [69], which also permits the use of some statistical tests (eg, randomization tests). Third, experimenter bias may occur when deciding whether conditions should be changed. As described above, the experimenter decides when to change conditions based on properties of the data path (eg, stability, sufficient number of data points). Some have suggested that the durations of conditions should be decided on an a priori basis [69]. This is certainly a possibility, but it means that the timing of a treatment condition may be inappropriate if the data are trending in the direction of the predicted treatment effect. Thus, the trade-off may not be desirable between reducing potential bias and decreasing the possibility of demonstrating experimental control through careful observation and decision making.

Another potential threat is the problem of small changes in the dependent variable as a result of an intervention. The threat concerns the conclusions the researcher may draw, or fail to draw, about the intervention. For example, a technology-based intervention may produce a small change in an outcome measure for only a fraction of participants. Relying on a stringent criterion, such as large visually detectable changes in graphically displayed data for all participants, to conclude that a treatment effect is present may result in a Type II error, or a false-negative. This may be especially problematic under two conditions [23]. First, if the intervention can be applied in a cost-effective way to a large number of individuals, a small behavior change may have considerable public health impact. Consider a simple, text-based motivational or cognitive-behavioral intervention for depression. If the intervention reduces symptoms in 2 of 6 participants in a single study, this may still be meaningful. This is because the intervention could be delivered to a large number of sufferers via mobile phones, so a 33% success rate in reducing symptoms may be important. (Of course, one single-case study showing such results would require replication(s) prior to larger-scale testing and dissemination). Second, if the outcome variable being measured is highly socially significant, a small reduction in behavior may also be meaningful. Consider a community-based intervention delivered via text to reduce suicide, domestic violence, or drinking and driving. Even a small reduction in any one of these outcomes would be important. Thus, if the scalability and/or social significance of the intervention are high, then the criterion to judge the clinical meaningfulness of the results will require special consideration.

Detecting small but meaningful changes in behavior can be accomplished using single-case designs. In addition to special consideration to criteria to judge treatment effects, special consideration must be given to the particulars of the research design. The researcher must choose designs (eg, multiple-baseline vs. reversal) and design parameters (eg, sufficiently long baseline and treatment conditions, sufficient number of participants to include in the study) to make detection of small but meaningful treatment effects more likely. In addition, detecting small but meaningful changes may be aided by statistical analysis [23,62].

## Dissecting Effects: Component Analysis of Technology-Based Interventions

A component analysis is “any experiment designed to identify the active elements of a treatment condition, the relative contributions of different variables in a treatment package, and/or the necessary and sufficient components of an intervention” [19]. Technology-based health interventions often entail more than one active treatment element. Determining the active elements may be important to increase dissemination potential and decrease cost. For example, a mobile health intervention to promote smoking cessation might entail two potentially active components: self-monitoring of progress plus access to on-demand therapeutic support from a counselor. Whether therapeutic support is necessary will have obvious dissemination and cost implications. Single-case research designs, in particular reversal and multiple-baseline designs, may be used to perform a component analysis. The essential experimental ingredients, regardless of the method, are that the independent variable(s) is systematically introduced and/or withdrawn, combined with replication of effects within and/or between subjects.

There are two main variants of component analyses: the dropout and add-in analyses. In a dropout analysis, the full treatment package is presented following a baseline phase, and then components are systematically withdrawn from the package. A limitation of dropout analyses is when components produce irreversible behavior change (ie, learning a new skill). Given that many technology-based interventions seek to produce sustained changes in health-related behavior, dropout analyses may have limited applicability. Instead, in add-in analyses, components can be assessed individually and/or in combination before the full treatment package is assessed. Add-in reversal or alternating designs “provide the most powerful and complete analysis of the active components of a treatment package because they reduce potential confounding from the effects of component combinations” [19]. Of course, the possibility of sequence effects should be considered, and researchers could address such effects through counterbalancing, brief “washout” periods, or explicit investigation of these effects [41].

**Figure 4:**
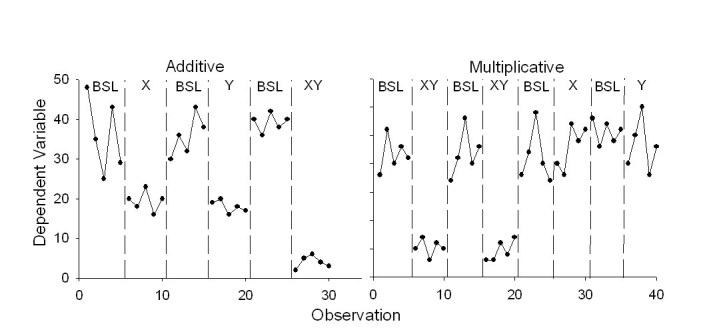
Two examples of possible results from a component analysis (BSL=baseline, X=first component, Y=second component).

Several conclusions can be drawn about the effects of the various technology-based components in changing behavior. The data should first be evaluated to determine the extent to which the effects of individual components are independent of one another. If they are, then the effects of the components are additive. If they are not, then the effects are multiplicative, or the effects of one component depend on the presence of another component. Figure 4 presents simplified examples of these two possibilities using a reversal design and short data streams (adapted from [19]). The panel on the left shows additive effects, and the panel on the right shows multiplicative effects. The data can also be analyzed to determine whether each component is necessary and sufficient to produce behavior change. For instance, using the example above, the panel on the right shows that neither the self-monitoring nor the counseling component is sufficient to promote cessation, and both components are necessary. If two components produce equal changes in behavior, and the same amount of change when both are combined, then either component is sufficient but neither is necessary.

The logic of the component analyses described here resembles new methodologies derived from an engineering framework [70,71]. During the initial stages of intervention development, these engineering-based methodologies use factorial designs to allocate participants to different combinations of treatment components. These designs, called fractional factorials because not all combinations of components are tested, represent excellent ways to screen promising components of novel technology-based treatment packages using randomized group designs. The components tested may be derived from theory or working assumptions about which components and combinations will be of interest. Collins and colleagues [70,71] note that such factorial designs may be more feasible in the field of technology-based health interventions relative to traditional in-person methods. The reason is that the costs of such interventions may be limited, for example when the costs are derived from the computer programming necessary to administer different treatment conditions. Once the programming is complete, delivering the appropriate version of the intervention across groups may be straightforward. Although this may be true in some cases, the costs (and other logistical issues—see above) associated with technology-based interventions are still formidable. Even a relatively small 16 condition fractional factorial may not be feasible [70]. Just as engineering methods seek to isolate active treatment components of novel interventions, so too do single-case methods. As such, they represent a viable alternative to isolate active components of technology-based interventions.

## Mechanisms of Behavior Change

During the past two decades, advances in statistical mediation analyses have allowed researchers to assess potential mechanisms of behavior change [72,73]. A mechanism refers to processes by which an intervention (or other independent variable) affects behavior. A drug might produce a behavioral effect via the mechanism of agonism or antagonism of particular receptors, and a technology-based behavioral health intervention might produce behavior change via specific mechanisms such as reinforcement, problem solving, or self-control. For example, a computer-based cognitive-behavioral treatment may produce drug abstinence via the mechanism of improved coping skills [74]. Although statistical mediation analyses provide evidence for the necessity of potential mechanisms of change, they do not provide evidence for the sufficiency of the relation between a potential mechanism and behavior change. As noted by Nock (2007), “just as correlation does not imply causation, mediation does not imply mechanism” (p. 5S [75]). Statistical mediation, therefore, is one step along the path of elucidating the necessity and sufficiency of a mechanism of behavior change. To our knowledge, statistical mediation approaches in single-case designs do not exist. Single-case experimental procedures, however, can be employed to isolate behavioral mechanisms. Therefore, single-case designs can help develop evidence on the necessity and sufficiency of a mechanism of technology-induced behavior change.

Several experimental criteria must be met to build a case for a mechanism of behavior change [75,76]. These criteria include gradient, temporal relation, and experiment. Gradient goes beyond showing an association (which can be accomplished using statistical mediation approaches) to showing that more of the treatment results in more of the mechanism and also more change in the outcome measure. Essentially, this is a parametric dose-response analysis, with the addition of measurement of potential mechanisms at each dose. For example, more exposure to a computer-based cognitive behavioral treatment [74] should result in more coping skills and therefore more drug abstinence. To achieve a temporal relation, an experiment must show that the change in the independent variable preceded a change in the mechanism, and the change in mechanism preceded the change in the outcome measure. This is where single-case procedures using technology are particularly well adapted: showing a temporal relation requires repeated, frequent assessment of the mechanism and outcome. The assessment can be enabled by technology-based approaches afforded by mobile phones, biometric sensors, or accessing a website. For example, mobile phones could be used to frequently probe changes in coping skills, and changes in skills should precede changes in drug abstinence. Finally, experiment means that researchers must use an experimental design that entails systematic manipulation of the independent variable (treatment). The mechanism should change only when the treatment is instituted and be temporally associated with changes in the outcome. Reversal designs or multiple-baseline designs, for example, can be used to meet the criterion of experiment.

Examining mechanisms of behavior change is crucial for understanding how technology-based interventions impact health outcomes (eg, increased activity, better dietary choices, sustained smoking abstinence, etc.). Isolating the key mechanisms can help ensure that these mechanisms are present when the interventions are scaled up and disseminated. This process may also increase the efficiency of an intervention by harnessing the active ingredients and discarding the inactive ingredients. Furthermore, isolating mechanisms can help bring parsimony to the field [75]. The number of technology-based interventions is multiplying, but a parsimonious assumption is that the number of mechanisms underlying these interventions is not keeping pace. Finally, because of technology’s unique ability to penetrate the daily life of the end-user, new mechanisms may be discovered and assessed. For example, technology-based therapeutic tools may be used in real-time, enabling “experiential learning”, which is an effective learning strategy that uses real-world interactions [77,78]. Overall, the symbiosis between technology-based assessment and the rigor of single-case designs suggests that we have an excellent opportunity to assess mechanisms of behavior change.

## Replication, Reproducibility, and Generality

Perhaps the most common concern with single-case research is its purported limited external validity or generality. Implicit in this concern is the premise that group designs deal with generality more effectively. Group designs, however, rarely include a random, representative sample of the relevant population and thus do not logically possess generality [21,79]. The problem of limited generality is even more likely in the context of initial efficacy testing, where groups may be based on convenience. Although some might assume that the issue of generality can be accommodated by inferential statistical testing, this is also a dubious assumption [80]:

A major limitation of statistical significance, therefore, is that it does not provide direct information about the reliability of research findings. Without knowledge about reliability there can be no examination of generality because repeatability is the most basic test of generality. Notwithstanding that limitation, however, significance testing based on group means may be seen, incorrectly, to have implications for generality of findings across subjects. Adherence to this view unfortunately gains strength as sample size increases. In fact, however, regardless of sample size, no information about intersubject generality can be extracted from a significance statement because no knowledge is afforded concerning the number of subjects for whom the effect actually occurred.

In a seminal article on null-hypothesis significance testing, similar considerations led Cohen to say, “For generalization, psychologists must finally rely, as has been done in all the older sciences, on replication” [49].

In the context of single-case research, generality can be demonstrated experimentally in several ways. The most basic way is via direct replication. Direct replication means conducting the same experiment on the same behavioral problem across several individuals (ie, a single-case experiment). For example, Raiff and Dallery [8] achieved a direct replication of the effects of Internet-based CM on adherence to glucose testing in 4 teens (as described earlier). One goal of the study was to establish experimental control by the intervention and to minimize as many extraneous factors as possible. Overall, direct replication can help establish generality across participants. It cannot answer questions about generality across settings, populations, or target behaviors. Instead, systematic replication can answer these questions. In a systematic replication, the findings from previous direct replication studies are extended to a new setting, population, or target behavior. The Raiff and Dallery study, therefore, was also a systematic replication of effects of Internet-based CM to promote smoking cessation to a new problem and to a new population because the procedure had originally been tested with adult smokers [11]. Effects of Internet-based CM for smoking cessation were also systematically replicated in an application to adolescent smokers using a single-case design [81].

By carefully choosing the characteristics of the individuals, settings, or other relevant variables in a systematic replication, the researcher can help identify the conditions under which a treatment works. To be sure, as with any new treatment, failures will occur. However, the failure does not detract from the prior successes: “…a procedure can be quite valuable even though it is effective under a narrow range of conditions, as long as we know what those conditions are” [82]. Such information is important for treatment recommendations in a clinical setting, and scientifically it means that the conditions themselves may become the subject of experimental analysis. This discussion leads to a type of generality called scientific generality [80], which is at the heart of a scientific understanding of technology-based interventions (or any intervention for that matter). As described by Branch and Pennypacker [80], scientific generality is characterized by knowledgeable reproducibility, or knowledge of the factors that are required for a phenomenon to occur. It can be attained through systematic replication and through analysis of behavioral mechanisms. Moreover, the data intimacy afforded by single-case designs can help achieve scientific generality about technology-based health interventions. That is, the fine-grained, replicated assessments of the ebb and flow of behavior can help us discover the mechanisms by which technology-based interventions affect health. Indeed, we know very little about theory-derived mechanisms by which these interventions affect health-related behavior [83]. Once we come to understand these mechanisms and the conditions under which they may be operative, they can be harnessed and tested in further studies and eventually be integrated into community-based interventions [44].

## Evolving Beyond Preliminary Efficacy

Although we focused on single-case experiments to establish preliminary efficacy in this paper, these designs can be used at all stages of technology-based treatment development [16]. For example, a series of single-case systematic replications can provide information about the efficacy and generality of an intervention [45], and recent methodological advances have promoted the use of single-case strategies for field-testing of interventions in naturalistic settings (ie, effectiveness research) [45,84]. Indeed, single-case experiments have generated a broad range of evidence-based practices in health care and related disciplines. These fields include clinical psychology [85], substance abuse [22,86], education [24], medicine [87], neuropsychology [25], developmental disabilities [23], and occupational therapy [88].

Single-case designs have similar promise to identify evidence-based practices in the field of technology-based health interventions. Because of their rigor and success rate in identifying evidence-based practices, some researchers have argued that highly controlled single-case designs should be considered on par with group designs (eg, randomized controlled trials) [87,89]. Rather than rank methods, we think it is more relevant for the researcher to have a diverse array of methodologies to choose from. Choosing the right method can be guided by several factors including logistics, experimental control, theory, and the previous education of the researcher [23]. We hope we have enhanced the last factor, and added some diversity to the ecology of research designs to test technology-based health interventions.
